# Measles outbreak in Dubrovnik-Neretva County, Croatia, May to June 2018

**DOI:** 10.2807/1560-7917.ES.2020.25.7.1900434

**Published:** 2020-02-20

**Authors:** Morana Tomljenovic, Mato Lakic, Tatjana Vilibic-Cavlek, Sanja Kurecic Filipovic, Vesna Visekruna Vucina, Andrea Babic-Erceg, Miljenko Ljubic, Iva Pem Novosel, Maja Ilic, Irena Tabain, Jelena Ivancic-Jelecki, Lisa Hansen, Bernard Kaic

**Affiliations:** 1 School of Medicine, University of Rijeka, Rijeka, Croatia; 2 European Programme for Intervention Epidemiology Training (EPIET), European Centre for Disease Prevention and Control, (ECDC), Stockholm, Sweden; 3 Croatian Institute of Public Health, Zagreb, Croatia; 4 Public Health Institute of Dubrovnik-Neretva County (PHIDNC), Dubrovnik, Croatia; 5 School of Medicine, University of Zagreb, Zagreb, Croatia; 6 University of Zagreb, Centre for Research and Knowledge Transfer in Biotechnology, Zagreb, Croatia; 7 Centre of Excellence for Virus Immunology and Vaccines (CERVirVac), Zagreb, Croatia; 8 National Institute for Public Health and the Environment (RIVM), Bilthoven, Netherlands

**Keywords:** airborne infections, communicable diseases, disease outbreak, viral infections, measles, vaccination, Croatia

## Abstract

In May 2018, measles was introduced in the Dubrovnik region by an adult who recently travelled to Kosovo*. Control measures and an outbreak investigation were implemented: 15 epidemiologically-linked cases met the outbreak case definition of a visitor/resident of Dubrovnik-Neretva County with laboratory-confirmed measles and symptom onset beginning on May 19. New cases were identified through hospitals and primary care physicians. Throat swabs, urine and/or serum samples were collected from outbreak cases. RT-PCR detection of viral RNA and IgM/IgG was used to confirm infection. The median age of cases was 33 years, with one 8 month-old infant. Vaccination status was unknown for 9 cases, three were unvaccinated, one case had history of one dose and two cases reported receiving two doses of measles-containing vaccine. There were 11 hospitalisations and one person developed pneumonia. Control teams undertook an extensive search of contacts and implemented a range of control measures. Despite the outbreak occurring at the beginning of the summer tourism season, it was contained and did not spread to neighbouring regions. With continuing measles transmission in Europe, even small outbreaks create a burden on the health system in countries which have eliminated measles, and illustrate the importance of maintaining high immunisation coverage.

## Background

In 2018, there was a large epidemic of measles in Europe with 83,540 cases of measles and 74 related deaths occurring that year [1]. The 2019 risk assessment of the European Centre for Disease Prevention and Control (ECDC) suggests that there is a high likelihood of further measles transmission among European countries [2]. Croatia’s neighbouring countries are experiencing ongoing measles outbreaks. In Serbia and northern Kosovo*, there were 5,798 reported measles cases and 15 deaths from October 2017 to August 2019 [3], in Bosnia and Herzegovina, 1,489 cases of measles were reported in 2018 and 2019, while in North Macedonia in 2018 and 2019, there were 1,948 cases of measles [4].

Childhood measles vaccination in Croatia is mandatory, free of charge and accessible through primary health care paediatricians for pre-school children and school medical specialists for school-aged children. The first dose of measles-mumps-rubella (MMR) vaccine is given at 12 months of age and a second dose is given to first grade school children from 5 to 7 years of age. The measles vaccine was introduced in Croatia in 1968 as a monovalent vaccine, and the combined MMR vaccine was introduced in 1976 [5].

**Figure 1:**
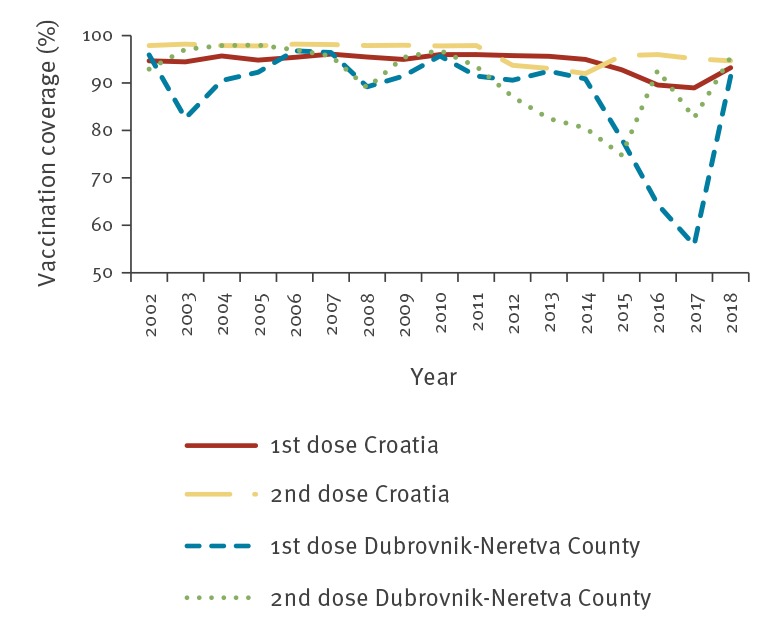
MMR vaccination coverage in Croatia and Dubrovnik-Neretva County, 2002–2018

Dubrovnik-Neretva County is the southernmost county of Croatia, with the city of Dubrovnik being the county seat. While the total county population is 121,381, Dubrovnik alone welcomed a record 1,271,657 tourists in 2018 [6]. MMR vaccination coverage for Dubrovnik-Neretva County steadily declined from 2014 to 2017, becoming the county with the lowest level of coverage in Croatia. In 2017, MMR first-dose vaccination coverage was 89% at the national level and 56% in Dubrovnik-Neretva County. Second-dose MMR vaccination coverage was 95% at the national level and 83% for this county (Figure 1).

MMR: measles-mumps-rubella.

The reasons for declining vaccination coverage are unknown. As in other parts of Europe, it is assumed that anti-vaccination sentiment has gained a greater audience, but there is no conclusive evidence to support this premise. Vaccination coverage estimates are based on reports from primary care physicians who check vaccination status annually for each vaccine-eligible child in their care.

## Outbreak detection

On 19 May 2018, an adult with a history of recent travel to Kosovo developed fever and subsequently visited three healthcare facilities in Dubrovnik-Neretva County, before developing a rash on 24 May. The patient was hospitalised in one of these facilities on 25 May and the hospital notified the Public Health Institute of Dubrovnik-Neretva County (PHIDNC) of a possible case of measles, which was confirmed on 29 May.

To prevent further spread of measles within Dubrovnik-Neretva County and to other counties, control teams were formed first at local level; additional personnel from the Croatian Institute of Public Health (CIPH) and other counties were involved at a later stage. On 6 July, the outbreak was deemed over; sixteen cases of measles were confirmed in Dubrovnik-Neretva County in this period.

This report presents the epidemiological investigation associated with this case and details the measures used to prevent further spread.

## Methods

### Case definition

A confirmed outbreak case was defined as a resident of or visitor to Dubrovnik-Neretva County, with laboratory-confirmed measles and symptom onset beginning on May 19. We used the European Union (EU) case definition for reporting of confirmed, probable and possible measles cases [7]. Following collection of laboratory data, suspected cases with clinical symptoms compatible with measles were categorised as confirmed, probable, possible or discarded (Box).

Box **Classification of cases during the measles outbreak in Dubrovnik-Neretva County, Croatia, May–June 2018**

**Confirmed case:** Any person meeting the clinical criteria (fever and maculopapular rash and one of the following: cough, coryza, conjuctivitis) with laboratory confirmation of measles (detection of IgM antibodies and/or RNA in clinical samples).

**Probable case:** Any person meeting the clinical criteria with an epidemiological link to a confirmed case.

**Possible case:** Any person meeting the clinical criteria without an epidemiological link to a confirmed case and without laboratory testing performed.

**Discarded case:** Any person meeting the clinical criteria with negative laboratory confirmation of measles.

### Case finding

Following confirmation of the first case on 29 May 2018, community healthcare providers and county hospitals were alerted about measles by the county epidemiologist. Since an outbreak of measles is defined by two linked cases [8], as soon as a second case was confirmed on 3 June 2018, the outbreak was identified.

Throughout the outbreak, healthcare facilities were urged to promptly report suspect cases to PHIDNC. Suspected measles cases (persons with clinical picture consistent with measles) were largely reported by hospital physicians and community practitioners to the PHIDNC by email and/or telephone, with PHIDNC immediately forwarding this information to the CIPH according to the routine reporting protocol.

Vaccination status was extracted by the PHIDNC epidemiologist from medical records, i.e. personal certificates of vaccination or immunisation provider records presented by the cases or their contacts; the epidemiologist did not rely on self-reported vaccination history. Additional exposure and demographic information was extracted from medical records and face-to-face interviews of cases and contacts. Age, sex, occupation, workplace, travel data, exposure information, symptoms and laboratory testing results were collected for all according to a routine, structured measles questionnaire. All data were stored at PHIDNC, while information on confirmed cases was forwarded to the CNIPH along with the official individual communicable disease notification form.

### Contact tracing

Persons who were in close contact with suspected cases during the infectious period, 4 days before and 4 days after onset of rash, were considered contacts. Contacts were identified by attending physicians and/or outbreak control teams led by epidemiologists who interviewed the cases. After the first secondary case was confirmed, control teams led by epidemiologists from PHIDNC were formed in order to manage the increased workload.

The outbreak control teams listed those who were in contact with the index case and secondary cases based on history of their movement during the infectious period. These lists included family contacts, friends and work colleagues who were in contact with the cases during the infectious period, patients visiting health facilities at the time cases attended the facilities and healthcare workers (HCWs) caring for cases during the infectious period. Control teams approached contacts, assessed their health status and risk for severe illness, checked vaccination status and provided immunisation per post-exposure protocol (i.e. if receipt of two doses of measles-containing vaccine was not documented, one dose was administered within 72 hours following exposure). Contacts were offered immunisation if they were unvaccinated or vaccinated with only one dose. When appropriate, control teams recommended self-isolation for 21 days following the last exposure, provided advice on the signs and symptoms of measles and provided instructions to immediately seek medical care in the event of symptoms. All identified contacts were successfully reached by telephone or door-to-door visits.

### Virological testing

Throat swabs, urine and/or blood samples were collected from suspected cases. Reverse transcription-PCR (RT-PCR) detection of viral RNA and IgM/IgG antibodies were used for laboratory confirmation of measles. Enzyme-linked immunosorbent assay (ELISA) IgM/IgG (Virotech Diagnostics, Rüsselsheim, Germany) and indirect immunofluorescence assay (IFA) IgM/IgG (Euroimmun, Lübeck, Germany) were used for serology. IgM-positive samples were additionally confirmed using IFA. According to World Health Organization (WHO) recommendation, serum samples were also tested for rubella IgM/IgG antibodies using ELISA (NovaTec Immunodiagnostica, Dietzenbach, Germany). In all cases, diagnosis was confirmed by detection of measles virus RNA from throat swab and/or urine according to the protocol described by Hummel et al [9]. Virology testing was performed at the WHO National Reference Measles/Rubella Laboratory at the CIPH in Zagreb. Genotyping for virus detected in the throat swab of the index case was performed at University of Zagreb, Centre for Research and Knowledge Transfer in Biotechnology.

### Ethical approval

Ethical approval for this study was not required since all activities are according to legal provisions defined by the Croatian Act on Protection of Population Against Infectious Disease [10].

## Results

### Descriptive epidemiology

**Figure 2:**
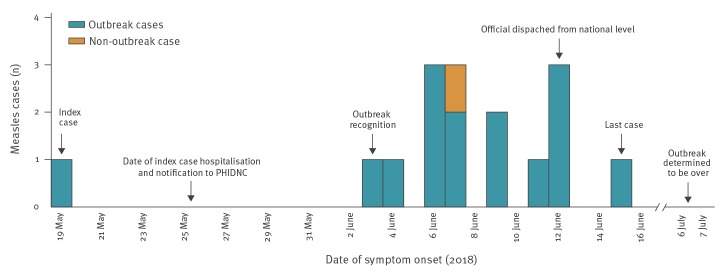
Confirmed measles cases in Dubrovnik-Neretva County by disease onset, Croatia, May–June 2018 (n = 16)

The index case developed symptoms on 19 May 2018, 3 days after returning from Kosovo. The last case had onset of symptoms on 15 June. In this period, there were 16 confirmed cases of measles in the county (Figure 2). One case was not epidemiologically linked to the index or other cases. The assumption was that this case had been exposed to measles in France where the case was working, and was therefore deemed a non-outbreak case. Of the 15 confirmed outbreak cases, 12 were in contact with the index case while two were attributed to secondary exposure. Laboratory results were obtained for all 13 discarded measles cases.

The median age of outbreak cases was 33 years (range: 8 months–56 years), with those 30 to 39 years of age most affected (7/15). There were eight male and seven female cases (Table). Eleven cases were hospitalised and one of these developed pneumonia. Four cases were HCWs and one was a student nurse, all of whom worked in the healthcare facility where the index case was hospitalised.

**Table:** Characteristics of measles outbreak cases in Dubrovnik-Neretva County, Croatia, May–June 2018 (n = 15)

Characteristics	Number
**Age group (years)**	
< 1	1
2–5	2
6–19	1
20–29	1
30–39	7
40–49	2
50–59	1
Median (range)	33 (8 months–56 years)
**Sex**	
Male	8
Female	7
**Country of infection**	
Croatia	14
Outside Croatia	1
**MMR vaccine status**	
2 doses	2
1 dose	1
Unknown	9
Unvaccinated	3

MMR: measles-mumps-rubella.

The index case had a history of travel outside of Croatia, while the location of infection for the remaining outbreak cases was in Dubrovnik-Neretva County.

Vaccination status was unknown for nine of 15 outbreak cases; of the other six cases, three, including the index case, were unvaccinated, one had documentation of a single dose of measles-containing vaccine and two had received two doses (Table).

From the 16 confirmed cases, i.e. the 15 outbreak cases and the one case with travel history in France, we identified 561 contacts. Of these, 168 were HCWs that were exposed in their workplace, 165 additional contacts were exposed in healthcare settings, mostly hospital. There were also 83 family contacts, and 145 other contacts from childcare facilities, schools and shopping centres. Of all contacts, 116, including 62 HCWs, received post-exposure vaccination. Three contacts, one immunocompromised HCW and two infants, received passive post-exposure prophylaxis.

Thirteen additional persons were initially identified as suspected measles cases. This led to the identification and follow-up of 150 contacts. These suspected cases were subsequently discarded as their laboratory tests for measles were negative. The discarded cases were mostly younger people, with a median age of 5 years (range: 1 month–57 years). Of the 150 contacts of discarded cases, 40 were HCWs, six were hospitalised patients, 71 were family contacts and 33 were contacts from childcare facilities, schools or shopping centres.

### Virological results

In all 16 cases, diagnosis was confirmed by detection of measles virus RNA in throat swab (n = 15) and/or urine (n = 8). Serum samples were collected from 13 cases. In two cases, measles IgM antibodies were found in serum samples and in one patient, seroconversion was documented in paired serum samples. Ten cases showed only measles IgG antibodies. The viral N450 sequence was submitted to the WHO Measles Nucleaotide Surveillance (MeaNS) database under the name MVs/Dubrovnik-Neretva.HRV/23.18/. The sample ID is 130457. The strain belongs to genotype B3 and its N450 sequence is identical to the sequence of the named strain MVs/Dublin.IRL/8.16/ [11].

## Outbreak control measures

Because this outbreak was linked to an index case who had visited healthcare facilities before clinical recognition of measles but during the period of infectiousness, additional control measures were implemented in healthcare facilities.

During the outbreak, 331 HCWs who lacked documented evidence of having received two doses of measles-containing vaccine were tested for anti-MV IgG. While 82.8% (n = 274) were immune, 12.4% (n = 41) tested negative and 4.8% (n = 16) were equivocal. In total, 395 HCWs without documentation of immunity, defined as receipt of two doses of measles-containing vaccine or serological evidence of immunity by detection of anti-MV IgG, were vaccinated. Some of the 41 HCWs who tested negative for anti-MV IgG were among the 395 vaccinated HCWs.

CIPH also provided several new guidelines and protocols for the prevention and control of measles outbreaks to the PHIDNC and health facilities in Dubrovnik-Neretva County, and to facilities in other counties on 12 June 2018. These guidelines pertained to the management of measles cases in healthcare facilities, the management of contacts, ensuring HCWs were immune to measles (i.e. had documented evidence of receipt of two doses of measles containing vaccine or anti-MV IgG seropositivity) and control measures for childcare facilities.

Multiple control measures were implemented in Dubrovnik-Neretva County, including contact tracing, post-exposure prophylaxis, isolation of cases and quarantine of contacts. At the beginning of this outbreak, the flow of people seeking care at the county hospital was rearranged in order to minimise contact between potentially infectious persons with other patients seeking care or being treated at the hospital. Non-immune HCWs were excluded from work, and susceptible children and staff were excluded from childcare facilities. Paediatricians were advised to invite parents of previously unvaccinated children over 12 months of age for vaccination in a catch-up campaign. In the catch-up campaign triggered by the outbreak, 898 children received vaccination from their healthcare providers. No specific measures were directed towards schools because the proportion of unvaccinated school children was lower than of preschool children, and the outbreak was detected in the last week of the school year.

The general public and the medical community received information about the outbreak, and control measures, from the CIPH and PHIDNC through various media. Information was offered about recognising measles symptoms, along with instructions to notify physicians by telephone before visiting health facilities. All parents were reminded about the importance of measles vaccination and urged to have unvaccinated children receive vaccine as soon as possible. HCWs were reminded that any person with a clinical picture consistent with measles should be immediately notified to a regional epidemiologist. The CIPH issued regular reports about the measles situation and during the outbreak, demand for serological testing for measles increased (data not shown).

In addition to the outbreak control team from Dubrovnik-Neretva County, other county-level control teams were established during the outbreak in order to support the control team if the outbreak cases increased in number or if the outbreak spread to other counties.

## Recommendations

Since the outbreak in 2018, the Croatian National Immunisation programme has recommended checking immunity to measles in HCWs working in specific healthcare departments, including infectious disease, hematology, paediatrics and neonatal care. It also recommended vaccination of HCWs without evidence of immunity at the beginning or during employment. As evidence of immunity to measles, a medical record documenting receipt of two doses of measles-containing vaccine, the first dose administered any time after 12 months of age and a minimum interval of one month between doses, or serological evidence of immunity is accepted. HCWs are advised that they will be excluded from work if they are unvaccinated and have contact with a measles case.

## Discussion

With the exception of 2014/15, when 220 measles cases were reported during an outbreak in Croatia, where the epidemic occurred in Roma population that was mostly unvaccinated [12], there are typically only a few cases of imported measles reported each year, without further transmission. In 2018, besides this small outbreak in Dubrovnik-Neretva County, there were seven imported cases reported in other counties, none of which resulted in further transmission of measles [13]. This is attributed to relatively high vaccination coverage.

The outbreak reported here lasted for 4 weeks and was deemed over on 6 July, 21 days (one maximum incubation period) after the onset of the last case’s symptoms on 15 June. Because of high media coverage and HCW awareness of measles virus circulation in the county, we did not expect any measles cases to be missed by the health system or unreported.

There was one case of measles-associated pneumonia. The typical hospitalisation rate for measles cases is one in four [14], and hospitalisation rates for other outbreaks in Europe ranged from 15.7% to 47% [15-18]. The high hospitalisation rate in this outbreak (11/15 cases) might reflect the need for isolation, rather than severe illness.

While all age groups were affected by measles in the EU/European Economic Area (EEA) from 2008 to 2017, the majority of cases (46–80%) were younger than 20 years of age [2]. In our outbreak, the median age was 33 years, with those 30 to 39 years of age most affected. There were no cases born before 1962. Persons born before 1961 in Croatia are considered to be immune to measles because of natural circulation of the measles virus during their childhood.

Prior to 2018 in Croatia, there were no statutory requirements for any specific occupational groups regarding MMR vaccination or immunity status. While all healthcare workers (HCWs) should have received vaccination as children [13], there is no routine check for measles immunity status before or during employment for HCWs. Vaccination status of cases is mostly unknown in adults in Croatia because of lost/missing medical documentation. For the four cases younger than 20 years of age, vaccination status was readily available, while vaccination status was determined from records for only two of 11 cases older than 20 years of age. Vaccination coverage in Dubrovnik-Neretva County when these cases were eligible for childhood vaccination ranged from 60% in the late 70s to 90% in the late 80s. Thus, some 10% to 20% of the most affected age group could have missed childhood vaccinations during a period when measles was not sufficiently prevalent to induce natural immunity [13]. Waning immunity cannot be excluded as a factor contributing to susceptibility among this age group and older cases.

We observed five cases of measles among HCWs, and 395 received measles vaccine during this outbreak, 62 as post-exposure vaccination and 333 as a part of control measure provided. There have been several outbreaks of measles in Europe among HCWs [19-21]. Since measles is highly contagious [22], HCWs can cause outbreaks or contribute to continued nosocomial transmission, which can jeopardise their own health and have direct impact on morbidity and mortality among their patients. The economic impact of controlling a measles epidemic can be considerable [23], especially in cases where HCWs are under-vaccinated.

The mobilisation of and increased public awareness of a potential infectious disease threat led to increased vaccination coverage for measles in Dubrovnik-Neretva County and across Croatia. The impact was seen immediately as first-dose MMR vaccine coverage in Dubrovnik-Neretva country went from 56% in 2017 to 91% in 2018. While there was an increase observed in vaccine uptake in early 2018, the outbreak in May 2018 undoubtedly contributed more than the early awareness campaigns to a stronger demand and increased vaccine uptake.

Although vaccination of children in Croatia is mandated by the Act on Protection of Population Against Infectious Disease, there has been a decline in vaccination coverage in the past decade. Parents who are hesitant towards vaccination can decline or postpone vaccination with minimal legal consequences. While Croatia’s neighbours, Serbia, North Macedonia, and Bosnia and Herzegovina have been dealing with large measles epidemics [3,4], Croatia has managed to control outbreaks and measles transmission thus far. Control of this small outbreak, and the final outcome of improved vaccination coverage, was accomplished through a massive effort of public health personnel and healthcare providers. Infectious disease transmission and the associated burden to individuals and the healthcare system could have been limited with continuous maintenance of high vaccination coverage, i.e. at least 95% for two doses of MMR.

### Conclusion

This outbreak was limited to 15 cases, and no deaths or disabilities were recorded. The outbreak did not spread to neighbouring counties, suggesting that the rapid outbreak control measures were effective. Notably, vaccine coverage among children in Croatia improved following the outbreak control intervention. With continuing measles transmission in Europe, even small outbreaks like this one create a large public health burden and illustrate the importance of maintaining high immunisation coverage.

## Note

*This designation is without prejudice to positions on status, and is in line with United Nations Security Council Resolution 1244/99 and the International Court of Justice Opinion on the Kosovo Declaration of Independence.
